# "Show me the money": a fair criticism of economic studies on inhaled bronchodilators for COPD

**DOI:** 10.1186/1471-2466-10-48

**Published:** 2010-09-15

**Authors:** Konstantinos Kostikas, Demosthenes Bouros

**Affiliations:** 1 Department of Pneumonology, Medical School, Democritus University of Thrace, Alexandroupolis, Greece

## Abstract

Chronic obstructive pulmonary disease (COPD) represents a significant burden for healthcare systems that is expected to grow further in the future. Inhaled long-acting bronchodilators, including tiotropium, represent the cornerstone of management of COPD patients. Economic studies evaluating the cost-effectiveness ratio of inhaled bronchodilators have to take into account several parameters, including the reduction of COPD exacerbations and related hospitalizations, as well as disease modification and improvement in quality of life and mortality. At an era when the healthcare resources are unlikely to grow as quickly as demand, economic analyses remain the cornerstone for the justification of the broad use of medication with an acceptable cost-effectiveness ratio. The greatest importance of such studies in COPD is the identification of subgroups of patients that will have the most benefit with an acceptable cost-effectiveness ratio for the healthcare providers. The development of models that will incorporate a global evaluation of the different aspects of this multi-component disease, in order to provide the best available care to each individual patient is urgently needed.

## 

Chronic obstructive pulmonary disease (COPD) represents a major cause of morbidity and mortality worldwide, with a significant economic burden for health systems and individual patients, both in high and in low income countries. According to the WHO Global Burden of Disease Project, COPD was the fifth leading cause of disease in 2001 and is expected to become the third leading cause by 2020 [1]. In this same report, COPD was also estimated to be among the ten leading causes of disability-adjusted life years in all countries [1]. The ageing of global population, along with the continued increase in the use of tobacco, especially in developing countries, represent the major causes for the expected increase in the burden of COPD [2]. The estimation of the overall cost of COPD for healthcare systems represents a major challenge, since both direct costs (including medication, medical equipment and hospital admissions) and indirect costs (including loss of work and productivity, disability and premature death) can be included [3]. Several confounding factors, including co-morbid diseases and socioeconomic differences, account for the reported variation in the annual cost of COPD between countries, ranging from as low as €151 for mild COPD in Italy to more than $ 10,000 for severe COPD in the U.S. [3,4]. It is now well-documented that a major contributor to the excessive cost of COPD for healthcare systems, especially in advanced disease, is due to the burden of COPD exacerbations (ECOPD) and especially the subsequent hospitalizations [5]. Most importantly, economic studies suggest that this burden of COPD may further grow in the future [6].

Inhaled long-acting bronchodilators represent the cornerstone of current management of COPD [7,8], providing improvement in significant patient-centered outcomes [9]. Several cost-effectiveness analyses of inhaled bronchodilators have been undertaken in order to justify reimbursement of those drugs by healthcare systems for the management of COPD patients, with contradictory results [10-14]. A 5-year cost-effectiveness analysis of tiotropium, salmeterol and ipratropium for the management of COPD in Spain suggested that tiotropium demonstrated the highest expected net benefit, if decision makers can afford to spend additional budget to gain additional health benefits, as expressed by quality-adjusted life-years [13]. In the accompanying paper of this commentary, Neyt and co-workers are to be congratulated for undertaking quite an amount of work in order to perform a cost-effectiveness analysis of the use of tiotropium in a large longitudinal observational database of patients from the Belgian Health System [15]. A model applying the relative treatment effect of the 4-year Understanding the Potential Long-term Impacts of Tiotropium (UPLIFT) trial on this population led to an unfavorable cost-effectiveness ratio for tiotropium, based on the reduction of the baseline risk of COPD exacerbation and exacerbation-related hospitalizations [15].

Differences in study design and definitions along with differences between healthcare systems may account for the observed differences between the study of Neyt et al. and previous ones, yet several factors have to be taken into account. COPD exacerbations represent a significant outcome in all major COPD therapeutic trials [16,17] and have a significant impact on mortality [18] and the outcome of individual patients [19]. Long-acting bronchodilators have been shown to reduce effectively COPD exacerbations and exacerbation-related hospitalizations [16,17], but this hardly represents the spectrum of improvements in everyday life of COPD patients that those agents provide. Tiotropium, specifically, has been shown to improve pulmonary function, dyspnea, exercise capacity and health-related quality of life in COPD patients, both in short-term [20,21] and in long-term [17] trials. There is additional evidence from post-hoc analyses of the UPLIFT trial that tiotropium reduces all-cause mortality and cardiovascular mortality and events in COPD patients[22], and may even reduce the rate of decline of FEV_1 _in patients with GOLD Stage II COPD [23], therefore modifying the natural history of the disease. Such outcomes are difficult to be incorporated in economic models. This is even more important since the proper definition of COPD exacerbations is still under debate between clinicians [24], and such events are highly underperceived and undertreated by individual patients [25].

Disease severity represents another important parameter that needs to be considered in the evaluation of efficacy of inhaled medication. The cost-effectiveness of tiotropium has been previously shown to be more favorable in more severe disease and in patients with previous hospitalizations [14]. In the study of Neyt et al. there was no analysis according to disease severity, yet the authors recognized that patients with previous hospitalizations for COPD exacerbations are the ones who may benefit from tiotropium treatment [15]. In a recent prospective economic analysis of a study that included patients with moderate-to-severe COPD with at least one exacerbation requiring medical intervention in the year before randomization, tiotropium proved to be the most cost-effective option, compared to the addition of salmeterol or salmeterol/fluticasone combination, despite a reduction in hospitalizations and an improvement in quality of life in patients receiving the combination treatment [10].

Besides the fact that Neyt and colleagues evaluated only a single outcome (i.e. the reduction in of exacerbations and exacerbation-related hospitalizations) in their cost-effectiveness analysis and they did not provide data according to disease severity, their study presents further limitations. The short-term horizon of one year chosen by the authors may not be enough for the appropriate evaluation of adverse events, given the low rate of COPD exacerbations per year reported in all the large trials [16,17], that was further confirmed in the Neyt study [15]. Moreover, the definition of this study for the use of tiotropium "on a more regular basis", including patients with ≥90 daily defined doses per year, suggests that several of the included patients were not compliant with their treatment. This is extremely important, since there is now evidence from the TORCH trial that adherence to inhaled drugs (as defined by >80% use of study medication) is associated with reduced risk of death and admission to hospital due to exacerbations in COPD [26].

In the last decade our understanding of COPD pathogenesis, natural history and treatment has improved significantly. A major part of this improvement is due to the fact that we have evaluated the effectiveness of treatments on patient-centered outcomes beyond the cornerstone measure of FEV_1_. In that time-course, we have learned that inhaled corticosteroids may reduce exacerbations [27], and even from the beginning there was a concern about high treatment costs with those drugs [28]. We have additionally learned that long-acting bronchodilators provide a lot more than bronchodilation, especially in the long-term [17], yet those drugs have a higher cost compared to short-acting bronchodilators [7]. Pulmonary rehabilitation has a favorable cost-effectiveness ratio [29], in contrast to lung-volume reduction surgery, even in highly selected patients [30]. Yet, all those interventions have contributed to an improvement in survival of COPD patients, that reflects overall better disease management in the last decade [31]. The main message from economic analyses remains the same: "the more you spent, the more you save". But where lays the thin line of cost-effectiveness? Probably somewhere between the lowest possible cost and the optimal treatment selection for each patient.

In a multi-component disease, such as COPD, many parameters have to be addressed in order to evaluate globally the individual patients and recognize possible therapeutic phenotypes [32]. Despite the fact that interventions aiming at the cost-effective early identification of new COPD cases [33] and the reduction of exacerbations will have the most impact on costs of COPD over the next 20 years [6], several other parameters, including comorbidities and quality of life, have to be constantly taken into account. At an era when the healthcare resources are unlikely to grow as quickly as demand, economic analyses remain the cornerstone for the justification of the broad use of medication with an acceptable cost-effectiveness ratio. The greatest importance of such studies in COPD is the identification of subgroups of patients that will have the most benefit with an acceptable cost-effectiveness ratio for the healthcare providers. What is urgently needed is the development of models that will incorporate a global evaluation of the different aspects of this multi-component disease, in order to provide the best available care to the individual patient.

## Competing interests

KK has received speaking fees and reimbursements for participation in congresses from Boehringer-Ingelheim and Pfizer, AstraZeneca, Nykomed, Novartis, GlaxoSmithKline and UCB Pharma and has participated in advisory boards held by Nycomed.

DB has received speaking fees and reimbursements for participation in congresses from Boehringer-Ingelheim and Pfizer, AstraZeneca, Novartis, GlaxoSmithKline and UCB Pharma and has participated in advisory boards held by Nycomed and Actelion.

## Authors' contributions

KK and DB have equally contributed to the conception and preparation of this commentary and they have both read and approved the final version of this manuscript.

## Pre-publication history

The pre-publication history for this paper can be accessed here:

http://www.biomedcentral.com/1471-2466/10/48/prepub
